# Anti-Oxidative and Anti-Apoptotic Oligosaccharides from *Pichia pastoris*-Fermented Cress Polysaccharides Ameliorate Chromium-Induced Liver Toxicity

**DOI:** 10.3390/ph17070958

**Published:** 2024-07-18

**Authors:** Imdad Ullah Khan, Aqsa Aqsa, Yusra Jamil, Naveed Khan, Amjad Iqbal, Sajid Ali, Muhammad Hamayun, Abdulwahed Fahad Alrefaei, Turki Kh. Faraj, Bokyung Lee, Ayaz Ahmad

**Affiliations:** 1 Department of Biotechnology, Abdul Wali Khan University Mardan, Mardan 23200, Pakistan; ik16092@gmail.com (I.U.K.); aqsaa3056@gmail.com (A.A.); yusrajamil22@gmail.com (Y.J.); naveedkhan@awkum.edu.pk (N.K.); 2 Department of Food Science and Technology, Abdul Wali Khan University Mardan, Mardan 23200, Pakistan; amjadiqbal@awkum.edu.pk; 3 Department of Horticulture and Life Science, Yeungnam University, Gyeongsan 38541, Republic of Korea; 4 Department of Botany, Abdul Wali Khan University Mardan, Mardan 23200, Pakistan; hamayun@awkum.edu.pk; 5 Department of Zoology, College of Science, King Saud University, Riyadh 145111, Saudi Arabia; afrefaei@ksu.edu.sa; 6 Department of Soil Science, College of Food and Agriculture Sciences, King Saud University, Riyadh 145111, Saudi Arabia; talasiri@ksu.edu.sa; 7 Department of Health Sciences, The Graduate School of Dong-A University, Busan 49315, Republic of Korea; bolee@dau.ac.kr

**Keywords:** antioxidants, apoptosis, chromium, fermentation, heavy metals, hepatotoxicity, oxidative stress, oligosaccharides, *Pichia pastoris*, reactive oxygen species

## Abstract

Oxidative stress impairs the structure and function of the cell, leading to serious chronic diseases. Antioxidant-based therapeutic and nutritional interventions are usually employed for combating oxidative stress-related disorders, including apoptosis. Here, we investigated the hepatoprotective effect of oligosaccharides, produced through *Pichia pastoris*-mediated fermentation of water-soluble polysaccharides isolated from *Lepidium sativum* (cress) seed mucilage, on chromium(VI)-induced oxidative stress and apoptosis in mice. Gel permeation chromatography (GPC), using Bio-Gel P-10 column, of the oligosaccharides product of fermentation revealed that *P. pastoris* effectively fermented polysaccharides as no long chain polysaccharides were observed. At 200 µg/mL, fractions DF73, DF53, DF72, and DF62 exhibited DPPH radical scavenging activity of 92.22 ± 2.69%, 90.35 ± 0.43%, 88.83 ± 3.36%, and 88.83 ± 3.36%, respectively. The antioxidant potential of the fermentation product was further confirmed through in vitro H_2_O_2_ radical scavenging assay. Among the screened samples, the highest H_2_O_2_ radical scavenging activity was displayed by DF73, which stabilized the free radicals by 88.83 ± 0.38%, followed by DF53 (86.48 ± 0.83%), DF62 (85.21 ± 6.66%), DF72 (79.9 4± 1.21%), and EPP (77.76 ± 0.53%). The oligosaccharide treatment significantly alleviated chromium-induced liver damage, as evident from the increase in weight gain, improved liver functions, and reduced histopathological alterations in the albino mice. A distinctly increased level of lipid peroxide (LPO) free radicals along with the endogenous hepatic enzymes were evident in chromium induced hepatotoxicity in mice. However, oligosaccharides treatment mitigated these effects by reducing the LPO production and increasing ALT, ALP, and AST levels, probably due to relieving the oxidative stress. DNA fragmentation assays illustrated that Cr(VI) exposure induced massive apoptosis in liver by damaging the DNA which was then remediated by oligosaccharides supplementation. Histopathological observations confirmed that the oligosaccharide treatment reverses the architectural changes in liver induced by chromium. These results suggest that oligosaccharides obtained from cress seed mucilage polysaccharides through *P. pastoris* fermentation ameliorate the oxidative stress and apoptosis and act as hepatoprotective agent against chromium-induced liver injury.

## 1. Introduction

Reactive oxygen species (ROS), such as superoxide anion, hydroxyl radicals, and hydrogen peroxide free radicals, play important roles in several cellular functions, especially in signal transduction and enzyme activity regulations (Rahal et al., 2014) [1]. However, due to their persistent over-production, ROS can interact with biological molecules producing by-products like peroxides and aldehydes that cause oxidative stress and damage cell architecture and function. Elevated levels of ROS have also been reported to cause DNA damage, which is considered the hallmark of apoptosis. In addition, elevated ROS is also responsible for the activation of several apoptotic pathways leading to cell death (Yesildag et al., 2022) [2]. Chromium (Cr) is among the leading wastes generated by various industries, including textiles, tanneries, electroplating, and metallurgical. The enormous production of waste Cr poses serious threats to both public and animal health. Various studies have reported that hexavalent chromium (Cr-VI) is comparatively more toxic as compared to trivalent chromium (Cr-III) (Shekhawat et al., 2015) [3]. Additionally, studies have also reported that the toxic Cr(VI) is comparatively more water soluble than Cr(III), making it more easily available to humans and animals (Chakraborty et al., 2022b, Chakraborty et al., 2022a) [4]. Several environmental pollutants primarily target the liver, as it is one of the main sites responsible for metabolism and blood detoxification. Subsequently, many ingested pollutants are absorbed into the bloodstream from the small intestine, which are then carried to the liver (Dong-Woo et al., 2016) [5].

Liver injury is a common pathological condition that can lead to fatty liver, cirrhosis, fibrosis, and even cancer. Multiple reports have confirmed that Cr causes hepatorenal toxicity by elevating the production of reactive oxygen species (ROS), resulting in damage from oxidative stress, apoptosis, DNA damage, genotoxicity, and carcinogenicity (Khalaf et al., 2020b) [6]. ROS are oxygen-containing reactive molecules that play a crucial role in cellular responses of signal transduction pathways in order to sustain life. However, excessive ROS production leads to organ and DNA impairment in various liver diseases and toxication (Sahreen et al., 2011) [7]. Thus, it is believed that antioxidant compounds may offer an effective and promising solution for the treatment of oxidative stress. Dietary supplementation of phytochemicals is widely applied as complementary and alternative medicines for the treatment of various disorders caused by oxidative stress. A previous study reported that methanolic extract of *P. stewartii* exhibited promising antioxidant, anti-inflammatory, and anti-cancer activities in diabetic rats (Rasheed et al., 2024) [8]. Polysaccharides are important biological molecules comprising monosaccharide units held together by glycosidic linkage (Qu et al., 2020) [9]. Polysaccharides from plants have gained much attention for their therapeutic potentials (Cao et al., 2018) [10]. Several natural polysaccharides have been proven to play significant roles in mitigating the oxidative stress induced by free radicals, thereby protecting the body from damage (Gao et al., 2022; [11] Zhu et al., 2023 [12]).

*Lepidium sativum* (commonly known as cress, garden cress, pepperwort and peppergrass), belonging to the family Brassicaceae, is extensively cultivated around the world, including the United States, Middle East, and Europe (Bisht et al., 2023) [13]. Extracts from cress have been extensively studied for various pharmacological properties, including hypoglycemic, hypotensive, bronchodilator, antimicrobial, and allelopathic (Ramadan and Oraby, 2020) [14]. Cress seed mucilage offers numerous therapeutic potentials in addition to its application as a food additive. Being hydrocolloid in nature, cress mucilage has shown promising antimicrobial and antioxidant properties. Notably, cress mucilage is cost-effective and widely employed in homemade remedies, which further enhances its versatility and utility (Al-Snafi, 2019) [15]. Oligosaccharides have been studied for their bioactive potential, rendering them valuable functional components in various industries including pharmaceuticals, food and cosmetics. Oligosaccharides trigger proactive responses to several biological complications, such as oxidative stress, inflammation and bacterial stress, either directly or through complex reaction cascades owing to their strong antioxidant and anti-inflammatory potentials (Sharma et al., 2022; [16] Su et al., 2023) [17]. Fermentation with *P. pastoris* is efficient and allows for the production of cost-effective oligosaccharides with consistent quality and well-characterized properties. Our previous study reported that *P. pastoris*-mediated fermentation of ethanol-precipitated polysaccharides produced oligosaccharides with strong antidiabetic potential (Khan et al., 2024) [18].

The present study was undertaken to produce bioactive oligosaccharides from cress seed mucilage polysaccharides through *P. pastoris*-mediated fermentation and to elucidate their antioxidant and hepatoprotective properties against chromium-induced hepatotoxicity in the albino mouse model.

## 2. Results

### 2.1. Polysaccharides Digestion via Yeast Fermentation

**Figure 1:**
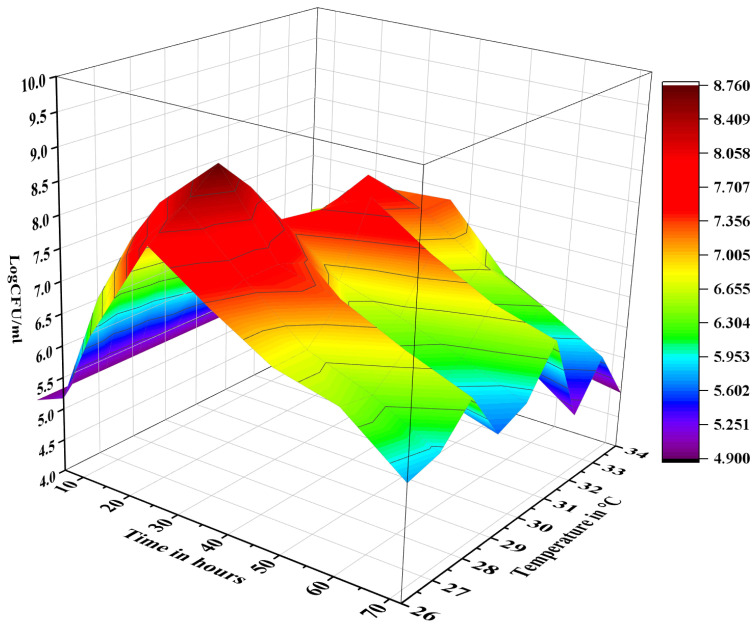
Growth phases of *P. pastoris* using cress seed mucilage polysaccharides as carbon source, where *Y*-axis shows *P. pastoris* count in logCFU/mL, *X*-axis shows time, and *Z*-axis shows temprature of the fermentation media.

The ethanol precipitated polysaccharides obtained from cress seed mucilage were in vitro fermented with *Pichia pastoris*. Fermentation media containing inulin and without any carbon source served as control. The growth of *P. pastoris* was monitored over 72 h, measuring the logarithmic colony forming unit per millilitre (logCFU/mL) at regular intervals (Figure 1). Specifically, the growth value of *P. pastoris* increased from 4.98 ± 0.13 logCFU/mL at 0 h to 7.76 ± 0.19 logCFU/mL after 6 h of fermentation. The *P. pastoris* cell count increased to 8.76 ± 0.17 logCFU/mL at 24 h mark, suggesting that EPP was effectively utilized as a carbon source. The growth of *P. pastoris* decreased to 7.93 ± 0.21 logCFU/mL after 36 h, 7.31 ± 0.11 logCFU/mL after 48 h, and 5.23 ± 0.16 logCFU/mL after 72 h. In contrast, no significant growth was recorded in the media where *P. pastoris* was grown in the absence of any carbon source.

### 2.2. Total Carbohydrates Contents

**Figure 2:**
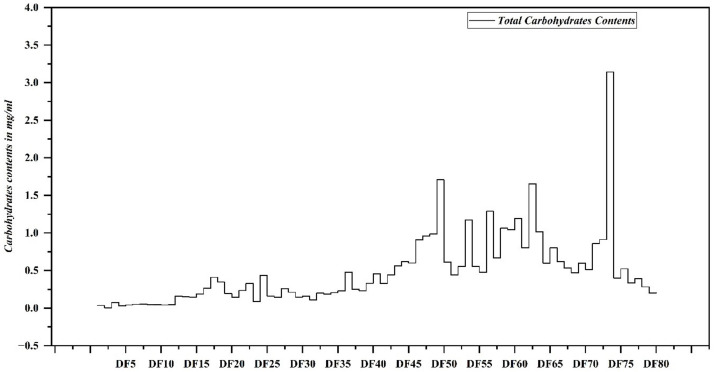
Total carbohydrate contents quantified in different oligosaccharide fractions were obtained through microbial bioprocessing of cress seed mucilage polysaccharides.

**Figure 3:**
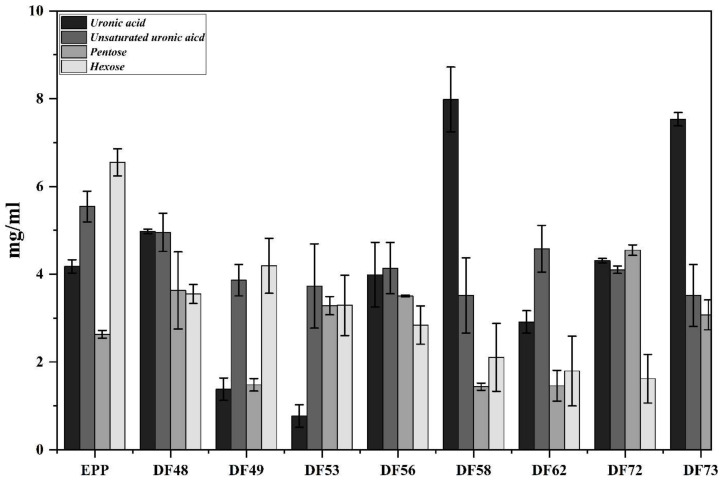
Biochemical composition of different oligosaccharide fractions obtained through microbial bioprocessing of cress seed mucilage polysaccharides.

The total carbohydrate content (TCC) of EPP fermented with *P. pastoris* and fractionated with gel permeation chromatography (GPC) was determined, and the results are expressed as ± mean mg/mL (Figure 2). The results revealed that among the fractions, DF73 contained the highest TCC of 3.14 ± 0.66 mg/mL, followed by DF53 (1.71 ± 0.32 mg/mL), DF72 (1.66 ± 0.23 mg/mL), DF62 (1.65 ± 0.27 mg/mL), DF56 (1.30 ± 0.01 mg/mL), DF60 (1.17 ± 0.69 mg/mL), and DF58 (1.06 ± 0.90 mg/mL). No long-chain polysaccharides were detected in fractions DF1 to DF29, suggesting that *P. pastoris* effectively utilized EPP as a carbon source. The selected oligosaccharide fractions were further screened for the presence of saturated uronic acid, unsaturated uronic acid, pentoses and hexoses, and the results obtained are illustrated in Figure 3. Results revealed that among the screened fractions, the highest saturated uronic acid was found in DF73 (7.98 ± 0.73 mg/mL), followed by DF72 (7.53 ± 0.05 mg/mL), DF53 (4.98 ± 0.05 mg/mL), and DF62 (3.99 ± 0.74 mg/mL). The highest unsaturated uronic acid was detected in DF53 (4.98 ± 0.05 mg/mL), whereas DF62 contained the least (0.77 ± 0.25 mg/mL). DF62 contained the highest pentoses (4.55 ± 0.12 mg/mL), followed by DF53 (3.63 ± 1.09 mg/mL), DF73 (3.28 ± 0.21 mg/mL), and DF72 (3.08 ± 0.34 mg/mL). Fraction DF53 contained the highest hexoses content (4.19 ± 0.63 mg/mL), followed by DF62 (3.55 ± 1.31 mg/mL), DF72 (3.29 ± 1.17 mg/mL), and DF73 (2.84 ± 0.44 mg/mL). This shows that varied amounts of saturated and unsaturated uronic acid, hexoses, and pentoses are present in fractions obtained after microbial digestions of polysaccharides.

### 2.3. Fourier Transformed Infrared (FT-IR) Spectroscopy of Fractions

**Figure 4:**
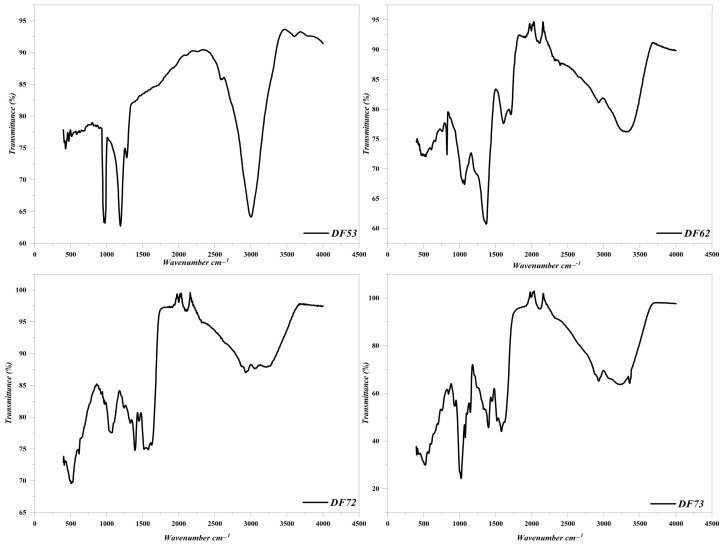
FT-IR spectroscopic analysis of Df53, DF62, DF72 and DF73 fractions.

In order to get insight into changes in the conformation of the constituent monosaccharide units and details of the glycosidic linkages present in the oligosaccharide fractions obtained through *P. pastoris*-mediated fermentation of EPP, FT-IR spectroscopic analysis of four fractions (DF53, DF62, DF72, and DF73) was carried out. Characteristic absorption peaks of oligosaccharides were observed at 3400 cm^−1^ and 2940 cm^−1^, which were attributed to O–H and C–H stretching, respectively (Figure 4). Additionally, stretching vibration at 1388 cm^−1^ was also attributed to the presence of the C–H group. The presence of these peaks confirmed the presence of carbohydrates in the fractions. Additionally, a strong absorption band was observed at 1610 cm^−1^, which indicated the presence of the ester group in oligosaccharide fractions, which further delineated its molecular structure and composition. A distinct peak in fraction DF73 was observed at 1615 cm^−1^, which could be attributed to the presence of the carboxylate group. Furthermore, notable bands at 750 cm^−1^ to 770 cm^−1^ confirmed the presence of β-type glycosidic linkage (Figure 4).

### 2.4. Mono- and Disaccharides Composition

The monosaccharide composition of the selected oligosaccharide fractions was confirmed using GC–MS (Table 1). Both acidic and neutral monosaccharides were detected in the selected fractions obtained after fermentation. Among them, galacturonic acid (71.23%) was found to be the most abundant monosaccharide in DF73. In DF53, the most abundant monosaccharide was galacturonic acid (32.18%), followed by glucose (17.32%), glucuronic acid (15.63%), and rhamnose (10.64%). Similarly, varied quantity of rhamnose was found in DF53 (10.64%), DF72 (3.03%), and DF73 (4.98%), while no rhamnose was detected in DF62.

### 2.5. Percent DPPH Free Radical Scavenging Activity of Oligosaccharides Fractions

**Figure 5:**
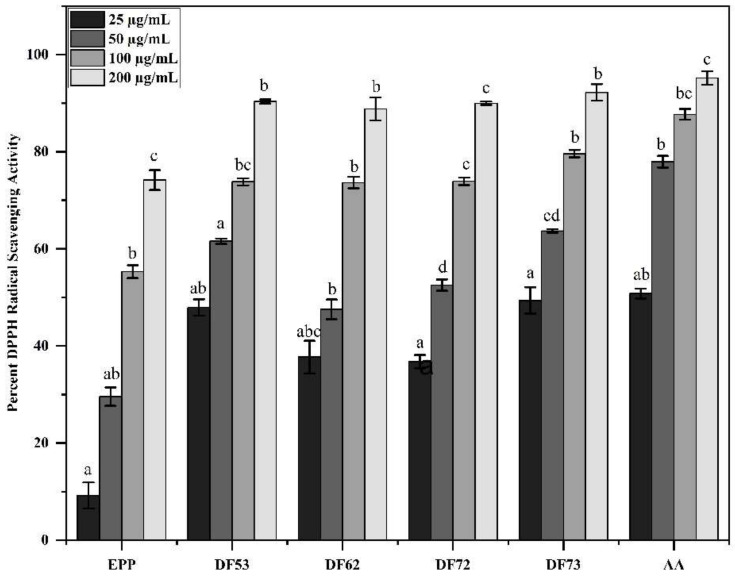
Percent radical scavenging of the DPPH free radical at different concentrations, where EPP; ethanol precipitated polysaccharides, DF53–DF73; oligosaccharides fractions and AA: ascorbic acid as reference compound. Means denoted by different letters (a, b, c…) are significant from each other at *p* value of 0.05.

Various studies have reported antioxidant potential of oligo- and polysaccharides. Here, we investigated the DPPH radical scavenging potential of selected oligosaccharide fractions and found that the oligosaccharides obtained as the product of fermentation exhibited stronger radical scavenging potential than EPP fractions (Figure 5). At 200 µg/mL, DF73 exhibited 92.22 ± 2.69% scavenging activity, while DF53 showed 90.35 ± 0.43%, DF72 displayed 89.97 ± 0.77%, DF62 demonstrated 88.83 ± 3.36%, and EPP showed 74.17 ± 1.06%. Ascorbic acid, which served as reference compound, scavenged 95.19 ± 3.03% of the DPPH free radical at 200 µg/mL. Similarly, at 100 µg/mL, ascorbic acid scavenged the DPPH free radical by 87.69 ± 1.13%, which was followed by DF73 (79.6 ± 0.74%), DF72 (73.92 ± 3.36%), DF53 (73.81 ± 0.73%), DF62 (73.64 ± 1.2%), and EPP (55.31 ± 1.32%).

### 2.6. Hydrogen Peroxide Radical Scavenging Potential

**Figure 6:**
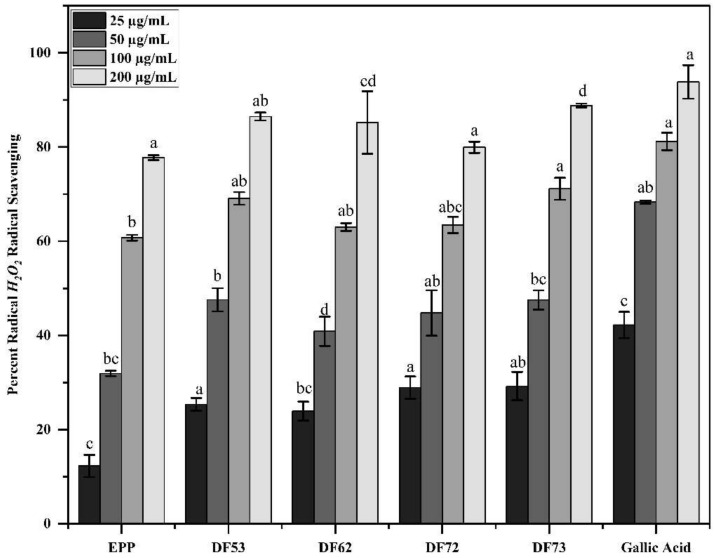
Percent radical scavenging of the H_2_O_2_ free radical at different concentrations, where EPP; ethanol precipitated polysaccharides, DF53–DF73; oligosaccharide fractions and Gallic acid: reference compound. Means denoted by different letters (a, b, c…) are significant from each other at *p* value of 0.05.

The percent hydrogen peroxide (H_2_O_2_) free radical scavenging assay was carried out to further establish the antioxidant potential of oligosaccharide fractions. Gallic acid was used as control. These fractions showed a dose-dependent H_2_O_2_ free radical scavenging activity (Figure 6). Among the tested fractions, at 200 µg/mL, the highest H_2_O_2_ radical scavenging activity was displayed by DF73, which stabilized the free radicals by 88.83 ± 0.38%, followed by DF53 (86.48 ± 0.83%), DF62 (85.21 ± 6.66%), DF72 (79.9 4± 1.21%), and EPP (77.76 ± 0.53%). The gallic acid (reference) showed 93.84 ± 3.57% activity at 200 µg/mL. Noticeably, here too, the radical scavenging activity of oligosaccharide fractions was higher than polysaccharides, indicating that *P. pastoris*-mediated fermentation produces stronger antioxidant saccharides. Table 2 shows the half-maximal inhibitory concentrations (IC_50_) in µg/mL, where gallic acid exhibited the most potent IC_50_ of 29.14 ± 2.28 µg/mL, indicating its strong radical stabilization potential. Among oligosaccharides fractions, DF73 displayed prominent IC_50_ of 51.63 ± 4.77 µg/mL, followed by DF53 (55.56 ± 0.36 µg/mL), DF72 (59.46 ± 5.32 µg/mL), DF62 (59.46 ± 5.32 µg/mL), and EPP (80.77 ± 0.01 µg/mL).

### 2.7. Effect of Oligosaccharides on Body and Liver Weight of Cr-Treated Mice

**Figure 7:**
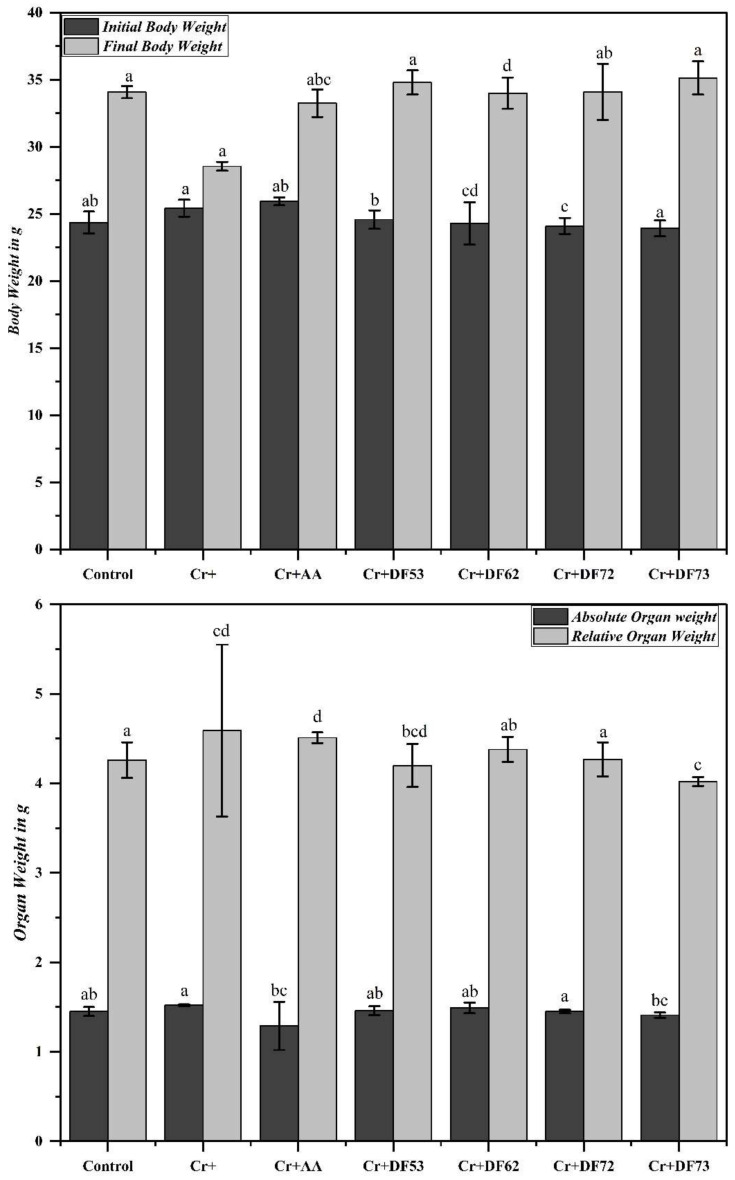
Initial and final body weight along with absolute and relative liver weight of the mice exposed to 100 mg/kg of Cr(IV) and co-treated with 200 mg/kg of oligosaccharides fractions. Means denoted by different letters (a, b, c…) are significant from each other at *p* value of 0.05.

Since the oligosaccharides exhibited strong antioxidant potential, we wanted to see the effect of oligosaccharides on the body and liver weight of experimental mice with and without Cr(VI) stress. A significant increase in growth was recorded in the normal controls, where the weight of the mice increased from 24.36 ± 0.82 g to 34.08 ± 0.45 g (Figure 7). The initial weight of the body across the group was relatively similar; however, the final body weight varied. The group of mice which received Cr(VI) showed an increase in growth, although not significant, as compared to the normal group, suggesting that Cr(VI) affects normal body growth. Interestingly, the body weight of the mice group which was exposed to 100 mg/kg Cr, along with 200 mg/kg of DF73, showed a significant increase in body weight, i.e., 23.93 ± 0.59 g initial body weight to 35.14 ± 1.24 g, which shows that oligosaccharides supplementation neutralizes the negative effects of Cr(VI) on the mice growth. We further noticed that Cr(VI) exposure results in an increase in liver weight, i.e., the absolute weight of the liver of normal mice were 1.45 ± 0.05 g, while that of the Cr-exposed group was 1.52 ± 0.01 g, probably due to inflammation caused by the Cr(VI)-induced stress. Treatment with ascorbic acid and different oligosaccharide fractions resulted in a lower absolute liver weight compared to Cr(VI) group. Among the groups, the absolute liver weight varied between 1.29 ± 0.27 g for ascorbic acid group, 1.41 ± 0.03 g for DF73, 1.45 ± 0.02 for DF72, 1.46 ± 0.05 g for DF53, and 1.49 ± 0.06 g for DF62. The relative liver weight (liver weight/body weight) showed a similar pattern, where the Cr(VI) group showed a higher relative weight, 4.59 ± 0.96, while that of the Cr(IV) + AA group was 4.51 ± 0.06. For the groups treated with different formulations, the relative liver weight ranged from 4.02 ± 0.05 for DF73 to 4.38 ± 0.14 for DF62.

### 2.8. Effect of Oligosaccharides on MDA Content in Cr(VI)-Exposed Mice

**Figure 8:**
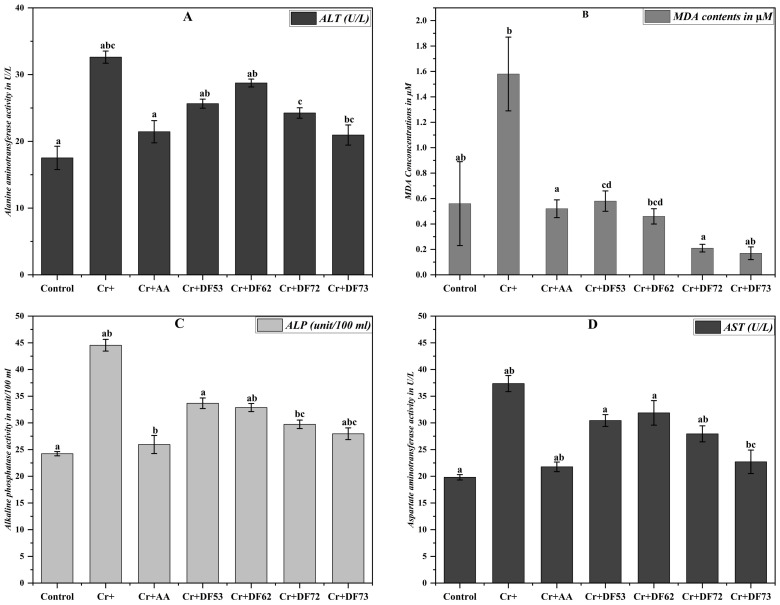
Effect of oligosaccharides on liver ALT, MDA, ALP and AST level of chromium exposed mice. Where (**A**) is ALT level in U/L, (**B**) is MDA level in µM, (**C**) is ALP level in units/100 mL and (**D**) is AST level in U/L. Means denoted by different letters (a, b, c…) are significant from each other at P value of 0.05.

The effect of Cr on the body and liver weight encouraged us to explore the effect of Cr exposure on malondialdehyde (Khan et al.) [19] production in the liver, which is an established biomarker for the assessment of oxidative stress. The MDA concentration (µM) in the liver of the mice exposed to Cr(VI) and those treated with different oligosaccharide fractions was monitored (Figure 8). Comparing the values, it is evident that Cr(IV) exposure induces strong oxidative stress in the liver (1.58 ± 0.29 µM MDA) as compared to the normal control (0.56 ± 0.33 µM MDA). Interestingly, treatment with Cr(VI), along with 200 mg/kg of different oligosaccharide fractions, led to decreased MDA concentrations ranging from 0.17 ± 0.05 µM for DF73, 0.21 ± 0.03 µM for DF72, 0.46 ± 0.06 µM for DF62, and 0.58 ± 0.08 µM for DF53. Similarly, mice exposed to Cr(VI), along with ascorbic acid as the positive control, resulted in the restoration of oxidative stress with an MDA concentration of 0.52 ± 0.07 µM, comparable to that of normal mice.

### 2.9. Effect of Oligosaccharides on Liver Function

Increased liver weight and MDA concentration indicated that Cr(VI) administration leads to oxidative stress in mice liver. Further, we decided to investigate the liver function of the experimental animal. For this purpose, we opted to monitor common marker enzymes used to assess liver function, i.e., ALT, AST, and ALP. Mice exposed to Cr(VI) exhibited significantly higher ALT concentration (32.62 ± 0.91 U/L) as compared to the control group (17.52 ± 1.75 U/L ALT), suggesting that chromium exposure induced liver damage in mice (Figure 8). Interestingly, treatment with ascorbic acid and oligosaccharide fractions resulted in lower ALT values compared to the Cr+ group, indicating a potential protective effect against chromium-induced liver toxicity. Among the oligosaccharide fractions, DF73 exhibited the lowest ALT level (20.95 ± 1.51 U/L), suggesting a particularly effective reduction in Cr(VI)-induced liver damage. Our results further revealed that Cr(VI) ingestion significantly elevated the ALP level in mice, indicating liver damage. However, treatment with ascorbic acid and oligosaccharide fractions lowered the ALP level as compared to Cr+ group, indicating hepatoprotection. The ALP level of Cr+ group mice was 44.54 ± 1.10 unit/100 mL, while that of the normal group was 24.23 ± 0.47 unit/100 mL (Figure 8). The ALP level of the mice group treated with DF73 was 27.96 ± 1.13 unit/100 mL, DF72 was 29.73 ± 0.86 unit/100 mL, and DF53 was 33.67 ± 1.28 unit/100 mL. Similarly, significant elevation was recorded in AST level in mice exposed to Cr(IV), i.e., 37.36 ± 1.5 U/L, as compared to the normal and ascorbic acid controls, where the AST levels were 19.81 ± 0.5 (U/L) and 21.77 ± 0.9 U/L, respectively. Oligosaccharides at 200 mg/kg promisingly stabilized the AST level where, among the screened oligosaccharide fractions, DF73 exhibited the lowest AST level (22.71 ± 2.2 U/L), followed by DF72 (27.95 ± 1.5 U/L), DF53 (30.44 ± 1.1 U/L), and DF62 (31.87 ± 2.3 U/L). Taken together, these results suggest that Cr(IV) induces strong oxidative stress in the mice liver which is then recovered by the potent antioxidant activity of the *P. pastoris*-fermented oligosaccharides from cress seed mucilage polysaccharides. 

### 2.10. Effect of Oligosaccharide Fractions on Liver DNA

**Figure 9:**
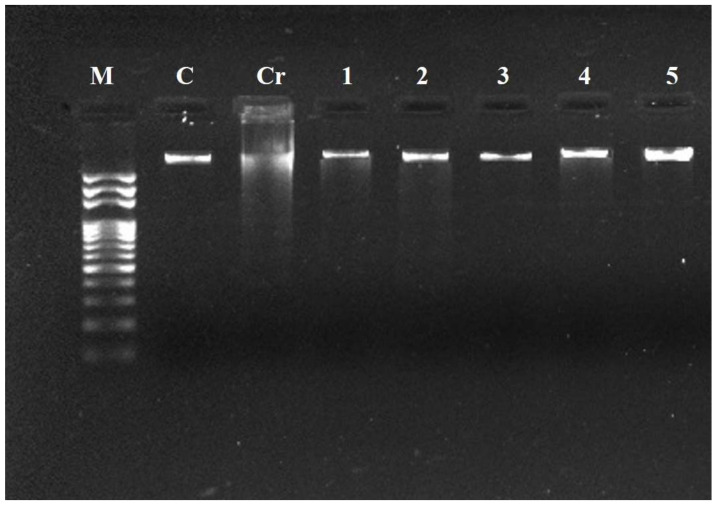
Oligosaccharides protect the DNA damage in mice liver induced by Cr (VI) exposure, where M; DNA marker (1 Kb), C: Normal mice, Cr: mice received 100 mg/kg of Cr (VI) alone, 1–4: mice received 100 mg/kg of Cr (VI) along with 200 mg/kg of DF53-73 oligosaccharides and 5; mice received Cr (VI) along with ascorbic acid.

Different heavy metals have been shown to cause DNA fragmentation. Next, we questioned whether the Cr(VI) treatment would affect DNA in the liver of experimental mice. It was noticed that the DNA extracted from mice treated with Cr(VI) only showed a smear when run on agarose gel, which showed that Cr(VI), like other heavy metals, caused DNA fragmentation (line Cr, Figure 9) as compared to line M, which served as the 1 Kb DNA ladder. The smear disappeared, and more intact DNA was seen when the Cr(VI)-treated mice were supplemented with oligosaccharides and ascorbic acid (positive control) (line 1–5, Figure 9), showing the ameliorating effect of oligosaccharides on DNA fragmentation caused by Cr(VI) toxicity.

### 2.11. Effect of Cr(VI) Toxicity and Oligosaccharides Supplementation on Liver Histology

**Figure 10:**
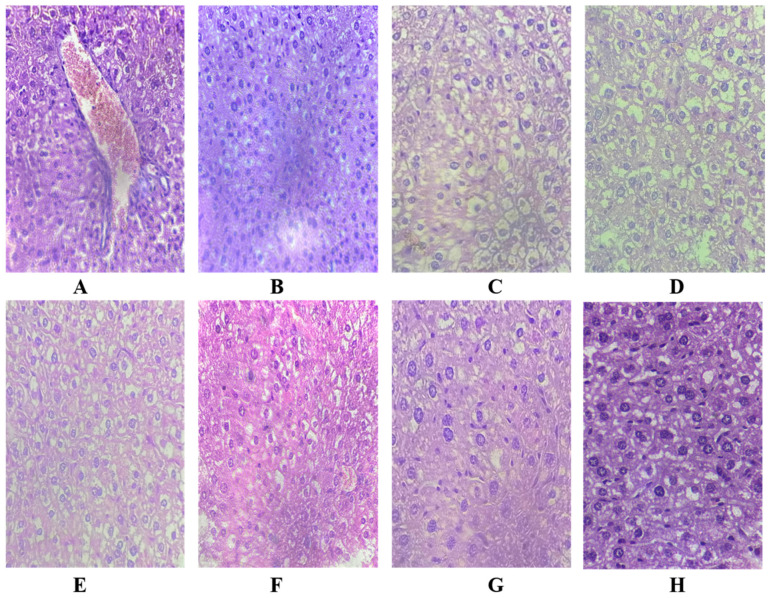
Histology of the mice liver stained with H&E stain: (**A**) received 100 mg/kg of Cr(VI) only, (**B**,**C**) normal control, (**D**) received 100 mg/kg of Cr(VI) + 200 mg/kg of DF53, (**E**) received 100 mg/kg of Cr(VI) + 200 mg/kg of DF62, (**F**) received 100 mg/kg of Cr(VI) + 200 mg/kg of DF72, (**G**) received 100 mg/kg of Cr(VI) + 200 mg/kg of DF73 and (**H**) received 100 mg/kg of Cr(VI) + 100 mg/kg of ascorbic acid.

We further investigated the effect of Cr(VI)-induced toxicity and the effect of oligosaccharides supplementation on liver histology in the experimental animals. It was observed that the normal mice that were not exposed to Cr(VI) displayed a well-organized hepatic structure characterized by normal sinusoidal spaces and normal morphology (Figure 10). In contrast, 100 mg/kg of Cr(VI) exposure induced observable hepatic damage, including the degeneration of hepatocytes, inflammation, vacuolization, and necrosis. Figure 10A shows that exposure to 100 mg/kg of chromium displayed diffuse vacuolization of hepatocytes cytoplasm, along with a swollen appearance of the necrotic cells compared to the normal cells (Figure 10B,C). Furthermore, Cr(IV) exposure also induced the disarray of the hepatic cord in most of the liver sections. However, upon treatment with 200 mg/kg of Cr + DF73, all the alterations induced by Cr(VI) exposure as portrayed in Figure 10G were improved. On the other hand, mice treated with Cr + DF73, Cr + DF72 (10E), and Cr + AA (10H) revealed a promising degree of improvement in hepatocyte structure and arrangement. Morphological analysis confirmed that treatment with oligosaccharides reduced the hepatocytes necrosis and ameliorated the structural disruption of hepatocytes caused by Cr(VI) exposure.

## 3. Discussion

Heavy metals pose serious threats to the ecosystem and public and animal health. They can accumulate in the food chain and infiltrate the human body through several routes, including the consumption of heavy-metal-polluted food, the inhalation of environmental pollutants, the drinking of contaminated water, and the exposure of the skin surface to harmful substances. Accumulation of higher concentrations of heavy metals such as chromium is toxic for living systems as it can interact with DNA and proteins thus inducing damage (Walker et al., 2012) [20]. Physio-chemical methods are commonly applied for heavy metal remediation; however, these methods are accompanied by high cost and side effects and are not so eco-friendly. There is a dire need to develop novel cost-effective and eco-friendly alternative approaches. Hexavalent chromium is listed as one of the most abundant bio-absorbable heavy metals that possesses toxic and detrimental effects to life (Ukhurebor et al., 2021 [21]; Ji et al., 2023) [22]. According to the International Agency for Research on Cancer Report (IARC), hexavalent chromium is classified as a group I occupational carcinogen. Exposure to Cr(VI) produces a significant amount of ROS, which induces oxidative stress and toxicity in various organs. This includes conditions such as anemia, asthma, failure of the liver, kidney, and reproductive system, and respiratory tract dysfunction (Balali-Mood et al., 2021) [23]. Further, oxidative stress caused by the overproduction of free radicals is widely accepted as the leading factor causing cell death.

Antioxidants are the molecules that have the ability to scavenge free radicals produced by the body as a result of oxidation–reduction reactions. Polysaccharides are complex carbohydrates made of repeating monosaccharide units and found abundantly in nature. Polysaccharides have garnered attention as effective antioxidants. Polysaccharides have been explored for their strong biosorbent affinity towards various toxic substances including heavy metals (Wu et al., 2022) [24]. Digestion of polysaccharides to oligosaccharides is mainly achieved by two methods, i.e., chemical and enzymatic methods. Chemical digestion offers several advantages, such as a simple procedure and low cost. However, it has drawbacks. The resulting digestion product is often intricate, posing a significant challenge for subsequent separation and purification. Additionally, this process can generate substantial pollution, posing serious threats to the environment (Chen et al., 2021) [25]. Enzymatic digestion of polysaccharides offers a compelling alternative due to its high specificity and mild reaction conditions. However, limitations like strict storage conditions, limited enzyme activity, difficulties in recycling, and high cost hinder its large-scale applications for oligosaccharides production (Xiao et al., 2017) [26]. In contrast, microbial digestion offers a one-step, cost-effective alternative to the large-scale production of bioactive oligosaccharides. *P. pastoris*, a methylotrophic yeast and a natural inhabitant of trees possesses a remarkable potential to utilize various carbon sources, including glucose, glycerol, and even methanol. Consequently, it has gained significant attraction as an effective host for the production of several commercial products, including recombinant proteins, enzymes such as trypsin and proteinase K, phospholipase C, and phytase (Rebnegger et al., 2016) [27].

In the current study, we evaluated the fermentation potential of *P. pastoris* using EPP as a carbon source extracted from cress seed mucilage. Our results demonstrated that *P. pastoris* effectively fermented EPP, and maximum growth was achieved after 36 h of fermentation. The fermentation of EPP resulted in the production of bioactive oligosaccharides, which were screened for their antioxidant and hepatoprotective potential. The monosaccharide composition of oligosaccharides extracted from *Codonopsis pilosula* was found to consist of glucose and fructose with a molar ratio of 1.21:1 (Bai et al., 2020) [28]. The monosaccharide composition of the oligosaccharides obtained from *Cimicifuga heracleifolia* was found to be 91.7% fructose and 8.3% glucose (Cui et al., 2023) [29]. The literature has shown that mucilage obtained from *Corchorus olitorius* has the highest uronic acid content, followed by galactose, rhamnose, and arabinose (Oh and Kim, 2022) [30]. The monosaccharide composition of polysaccharides extracted from *Opuntia ficus-indica* gum showed the presence of similar types of monosaccharide units as reported in our study but with different concentrations (Ginestra et al., 2009) [31]. Similarly, polysaccharides extracted from *Lepidium perfoliatum* seed mucilage were shown to contain rhamnose, galactose, arabinose, glucose, and xylose in variable quantities (Koocheki et al., 2022) [32]. Previous studies reported that oligosaccharides extracted from Anax ginseng contain a broad stretching at 3401 cm^−1^ to 3408 cm^−1^, which is assigned to the hydroxyl group (Zhao et al., 2020) [33]. The FT-IR spectra of oligosaccharides gave the first excited vibration, which could be separated into two band ranges—the fingerprint region and the straight-line region. The fingerprint region of the FT-IR of oligosaccharide comprised a 1200 to 900 cm^−1^ region, which was fundamentally associated with the stretching vibration of C–C, C–O, and C–O–C, while no information can be elucidated from the straight-line region. Our results revealed the presence of various characteristic functional groups of carbohydrates (Amanah et al., 2022) [34]. Similarly, the band at 1647 cm^−1^ could be attributed to the absorption of water, while the band which was present at 1058 cm^−1^ indicated the presence of C–O stretching (Bai et al., 2020) [28].

Persistent overproduction of ROS and reactive nitrogen species (RNS), generated from both endogenous and exogenous sources, are responsible for causing oxidative stress. On the other hand, antioxidants are compounds that play a significant role in delaying, controlling, and inhibiting the process of oxidation (Jomova et al., 2023) [35]. The antioxidant potential of natural compounds can be evaluated using various in vitro antioxidant assays. In this study, we evaluated the antioxidant potential of oligosaccharide fractions obtained from the microbial digestion of EPP from cress seed mucilage using DPPH free radicals. Previous studies have shown that the antioxidant activity of polysaccharides was related to their uronic acid content, monosaccharide composition, molecular weight, and the type of glycosidic linkage (Zhao et al., 2020) [33]. The antioxidant potential of tea polysaccharides has been directly related to their uronic acid content as different concentrations and their composition could affect the antioxidant properties. Furthermore, high uronic acid content in polysaccharide fractions had been demonstrated to have greater free radical scavenging activities. In the DPPH assay, DPPH would lose absorbance by combining an odd electron from hydrogen- or electron-donating compounds via hydrogen atom transfer or single electron transfer (Hung et al., 2021) [36]. Similarly, the antioxidant potential of okra pecto-oligosaccharides was higher than pecto-polysaccharides because of their low molecular weight (Yeung et al., 2021) [37]. H_2_O_2_ is a highly reactive free radical which can easily penetrate through the cell membrane and can react with intracellular ions thus leading to cellular damage. The accumulation of free radicals in the cells has been identified to be the root cause of many clinical disorders, including aging, inflammation, diabetes, cardiovascular, cancer, and obesity (Sadasivam et al., 2022) [38]. Previous studies have reported that polysaccharides exhibit promising H_2_O_2_ radical scavenging potential and thereby attenuate their detrimental effects (Alkahtani et al. [39], 2020; Yi et al., 2023) [40]. The H_2_O_2_ radical scavenging capabilities of oligosaccharide fractions may be attributed to its proton-donating potential. The C_2_ and C_6_ of the oligosaccharides are generally responsible for H-atom transfer towards the free radicals, thereby stabilizing them (Cheong et al., 2022) [41]. Therefore, it can be hypothesized that hydrogen abstraction may occur in oligosaccharide fractions obtained from microbial digestion of EPP to yield a more stable H_2_O_2_–oligosaccharide complex.

A previous study reported that Cr(VI) exposure in mice did not exert any observable effect on their body weight (Shil and Pal, 2019 [42]; Zhang et al., 2024) [43]. In our study, we found that the weight of the mice increased in all the experimental groups. However, the mice group which were exposed to Cr(VI) treatment and supplemented with oligosaccharides exhibited significant weight gain as compared to the group which received Cr(VI) treatment only. Conversely, it was found that the Cr(VI) exposure increases the liver weight of the mice. Cr(VI) exposure can inflict damage on the liver, causing lesions, chronic inflammation, elevated production of Kupfer cells, and fatty degeneration. These detrimental effects could lead to an enlargement of the liver, which would result in the observed gain in weight in the mice liver (Suljević et al., 2021) [44]. Similarly, our results concluded that weight gain in the mice liver was observed in the Cr(VI)-treated group. However, the mice group which was treated with Cr(VI) coupled with different oligosaccharides fractions did not exhibit any significant weight gain in the mice liver. It could be concluded that the oligosaccharides exert hepatoprotective effects and thereby mitigate the adverse effects caused by chromium stress. ALT, ALP, and AST are liver enzymes, and an elevated level of these enzymes would indicate liver damage. Previous studies have reported that a persistently elevated level of ROS is the root cause of increasing levels of ALT, ALP, and AST. ROS can damage cells, including liver cells. Damage to the liver produces and secretes these enzymes into the bloodstream, which is a sign of hepatotoxicity. There is a potential link between oxidative stress and elevated levels of these enzymes (El-Demerdash et al., 2021) [45].

Various in vitro and in vivo studies have demonstrated that Cr(VI) triggers oxidative stress by increasing the generation of ROS, resulting in the degradation of lipids and enzymes and DNA damage. Within the cell, a cascade of cellular events occurs in response to Cr(VI)-mediated oxidative stress, encompassing the increased production of superoxide anions and hydroxyl radicals. Furthermore, Cr(VI)-mediated oxidative stress also results in enhanced lipid peroxidation, activation of protein kinase C, DNA fragmentation, changes in gene expression, modulation of intracellular oxidized states, and apoptotic cell death. ROS initiates the breakdown of the DNA double-strand, thereby activating the apoptotic pathway; consequently, a cascade of apoptosis occurs (Lv et al., 2018) [46]. It has been reported that elevated levels of ROS cause oxidative damage to hepatocytes, which is characterized by sinusoidal dilation, drastic architectural disruption in parenchyma, and central vein congestion (Suchana et al., 2021) [47]. Similar observations were recorded in our study, where Cr(VI) uptake caused significant sinusoidal dilation, vascular congestion, hemorrhaging, necrosis, and distortion in hepatocytes, which eventually resulted in degenerative changes. However, our results concluded that oligosaccharides supplementation can reverse the damage caused by Cr(VI) to the liver.

## 4. Material and Methods

### 4.1. Chemicals and Equipment

K_2_Cr_2_O_7_ (99% purity) as a source of Cr(VI) and 1, 1-diphenyl-2-picrylhydrazyl (DPPH) were purchased from Sigma-Aldrich (Steinheim, Germany). Bio-Gel P-10 was obtained from Bio-Rad Laboratories (Hercules, CA, USA). All the chemicals used in this study were either of HPLC grade or analytical grade. Stock solution of the Cr(VI) and different screening compounds were prepared in autoclaved distilled water. All the working solutions were freshly prepared prior to the experiments.

### 4.2. Polysaccharides Extraction

The mucilage was extracted by soaking cress seeds in sterilized distilled water, as described previously (Karazhiyan et al., 2011) [19]. Briefly, 20 g of cress seeds were soaked in 1000 mL of distilled water and left overnight at room temperature. The aqueous mixture was then filtered using muslin cloth to isolate the mucilage. Polysaccharides were obtained by treating cress seed mucilage with 75% (*v*/*v*) ethanol overnight on a shaker. Precipitated polysaccharides were collected by centrifugation at 4000 rpm for 30 min. The supernatant was discarded, and the pellet containing polysaccharides was lyophilized. The obtained polysaccharides were confirmed using the phenol sulfuric method, as mentioned in Section 2.5, and were stored at −20 °C for further use.

### 4.3. In Vitro Fermentation Using Yeast

The free inoculum of the yeast (*Pichia pastoris*) was cultured in YPD (yeast extract, peptone and dextrose) media for 24–48 h, and the cells were counted manually using a Neubauer chamber under a light microscope. Further, 1 × 10^5^ yeast cells/mM were added to fermentation media containing 1% ethanol precipitated polysaccharides as the carbon source and ammonium chloride (NH_4_Cl) as the nitrogen source. The fermentation reaction was incubated in a shaking incubator at 28–30 °C, and yeast growth and pH of the fermentation media were monitored for different time intervals, i.e., 0, 6, 12, 24, 36, 48, 72, and 96 h. After 96 h of fermentation, the yeast cell biomass was separated from the fermentation media using a centrifuge at 4500 rpm for 25 min. The supernatant was collected in a fresh, sterilized tube, and the pellet was discarded.

### 4.4. Gel Permeation Chromatography

Size-based purification of oligosaccharides obtained through microbial digestion of EPP was performed through Gel Permeation chromatography (GPC) using Bio-Gel P-10 column. Briefly, 5 mL aliquot of ferment product was loaded to the column and eluted with distilled water at a flow rate of 0.3 mL per min. A total of 90 fractions (2 mL each) were collected for further analysis.

### 4.5. Biochemical Analyses

The total sugar content in the oligosaccharides was determined using the phenol sulfuric acid method (Masuko et al., 2005) [48]. The uronic acid contents were determined by the m-hyrdoxy biphenyl method (Blumenkrantz and Asboe-Hansen, 1973) [49]. Unsaturated uronic acid content was quantified using the thiobarbituric acid method (Payasi and Sanwal, 2003) [50]. Hexose content was determined by the anthrone reagent method (Dische, 1962) [51]. Finally, the pentose content in oligosaccharide fractions was determined by the orcinol solution method (Dische, 1962) [51].

### 4.6. Monosaccharides Composition Analysis2

The monosaccharide composition analysis of the selected oligosaccharide fractions was carried out using gas chromatography–mass spectrometry (GC–MS) (Pettolino et al., 2012) [52]. Initially, 0.2 mg of each sample was hydrolyzed with 4 mL of 2 M trifluoroacetic acid for 4 h at 110 °C. Excess TFA was removed by evaporation at 45 °C, followed by methanol treatment. The resultant residues were filtered through a 0.22 µm filter, and the filtrate was subjected to GC–MS analysis using a Dionex ICS system coupled with CarboPac PA1 guard column (50 × 2 mm). Known amounts of fucose, D-glucose, xylose, mannose, galactose, arabinose, and glucuronic and galacturonic acid were used as standards (Lee et al., 2021) [53].

### 4.7. Fourier Transformed Infrared (FT-IR) Spectroscopy

The functional groups present in the selected oligosaccharide fractions, including DF53, DF72, and DF73, were characterized through FT-IR spectroscopy using a Bruker and Tensor spectrometer from Germany. Briefly, a tablet was made by mixing 2 mg of oligosaccharide samples with dried potassium bromide (KBr), and the spectra were then recorded in the range of 4000 to 400 cm^−1^.

### 4.8. Antioxidant Activity via DPPH Assay

The antioxidant potential of the oligosaccharide fractions was evaluated using the DPPH radical scavenging assay (Ji et al., 2020) [54]. The fractions were screened at 50, 100, and 200 µg/mL concentrations. Each sample was mixed with 4 mM of DPPH solution, vortexed, and incubated in the dark for 30 min. Ascorbic acid served as the standard. The absorbance was measured at 517 nm. The percentage of DPPH radical scavenging activity was calculated using the following formula:

$$\%\;\mathrm{DPPH}\;\mathrm{radical}\;\mathrm{scavenging}\;\mathrm{activity}=\frac{\left(A0-A1\right)}{A0}\times 100$$

where A0: absorbance of control, A1: absorbance of the sample.

### 4.9. Hydrogen Peroxide Radical Scavenging Assay

The hydrogen peroxide (H_2_O_2_) radical scavenging activity was performed in vitro, where the reduction in the absorbance of H_2_O_2_ signified its oxidation (Shao et al., 2020) [55]. A solution containing 43 mM of H_2_O_2_ was freshly prepared in 0.1 M phosphate buffer (pH = 7.4). Phosphate buffer (3.4 mL) and H_2_O_2_ (0.6 mL) were mixed in a reaction tube, and the oligosaccharides were added, along with gallic acid, to different screening concentrations. The reaction was incubated in the dark for 25 min at room temperature, and the absorbance was recorded at 230 nm. A solution containing phosphate buffer and lacking H_2_O_2_ served as a blank. The percent scavenging activity of each sample was calculated using the following equation:

$$\%\;Scavenging\;activity=\left(1-\frac{As}{Ac}\right)\times 100$$

where *As* is absorbance of sample and *Ac* is absorbance of control.

### 4.10. Hepatotoxicity and Cytoprotective Studies in Experimental Mice

Balb/C albino mice were acquired from the Veterinary Research Institute (VRI), Peshawar, Pakistan. The animals were acclimatized in a controlled environment and maintained under standard conditions, with access to food and water. Animal handling was carried out according to the guidelines documented by the National Institute of Health (NIH) (Washington, DC, USA) for the care and use of lab animals. All the experiments performed in this research work were approved and performed according to the guidelines provided by the ethical committee of the Department of Biotechnology, Abdul Wali Khan University Mardan, Khyber Pakhtunkhwa, Pakistan (Certificate Approval number No: AWKUM/Biotech/2023/2785). After two weeks of acclimatization, the animals were divided into twelve groups of 5 mice in each group. Group-I (normal control) was administered with water only, Group-II received oral administration of 100 µL of potassium dichromate at a concentration of 100 mg/kg, and Group-III received co-treatment of 100 µL of potassium dichromate (100 mg/kg) and 100 µL of ascorbic acid (100 mg/kg). Group-IV to XII received 100 µL of potassium dichromate (100 mg/kg) coupled with 100 µL of DF53, DF72, and DF73 at a concentration of 200 mg/kg. The experiment was carried out for 21 days; after that, the mice were fasted for 12 h and then euthanized using diethyl ether (Khalaf et al., 2020a) [4]. All the experiments were repeated in triplicate.

### 4.11. Body and Organ Weight

The body weight of the experimental mice was recorded at the beginning (initial weight) and end (final weight) of the treatment. The livers were dissected, and the attached tissue were trimmed off and weighed. Weight of the liver was expressed as g/100 g of the body weight.

### 4.12. Biochemical Assessments of Liver

The liver samples were first frozen and then thawed to 4 °C. After thawing, the tissue was homogenized in 1.15% potassium chloride (KCl) using a homogenizer. The homogenized solution was centrifuged at 9000 rpm for 15 min at 4 °C, and the supernatant was collected. The malondialdehyde (Khan et al.) [18] was determined using the thiobarbituric acid reaction assay (Ohkawa et al., 1979) [56]. The optical density of the solution was recorded at 540 nm, and the results were expressed as ± mean percent reduction in MDA contents.

### 4.13. Screening of the Liver Function Enzyme

The aspartate aminotransferase (AST), alanine aminotransferase, and alkaline phosphatases (ALP) levels in the liver homogenate were measured using commercially available kits.

### 4.14. DNA Fragmentation Assay

DNA was extracted from the liver tissue of the experimental mice using the phenol–chloroform–sulfuric acid method. The DNA extracted from each sample was mixed with 5 µL of loading dye (bromophenol blue) and loaded into 2% agarose gel containing 0.5% ethidium bromide. A laddering pattern of the DNA was determined by running the gel at 70 V for 30 min using gel electrophoresis assembly (Bio-Rad Laboratories, Hercules, CA, USA). Gene Ruler 1 Kb DNA marker (Thermo-fisher Scientific, Waltham, MA, USA) was used as a standard DNA ladder. The DNA fragmentation was visualized using a Gel-Doc (Bio-Rad Laboratories).

### 4.15. Histopathological Observations

The liver of the experimental mice was fixed in 10% (*v*/*v*) formalin and embedded in paraffin wax (Li et al., 2019) [57]. Further, 5 µm sections were made and processed for histological analysis using hematoxylin and eosin staining. The sections were photographed using a light microscope.

### 4.16. Statistical Analysis

Data from different groups were expressed as mean ± standard deviation. Data analysis was performed using various software tools, including Statistical Package for the Social Sciences (SPSS 6.1), Microsoft Excel 2007, and GraphPad Prism (version 8). Comparison between the groups was performed through one-way ANOVA followed by Tukey’s post hoc test. *p* value ≤ 0.05 was considered to be significant.

## 5. Conclusions

Oligosaccharides were obtained from EPP through microbial digestion with *P. pastoris*. Conditions were optimized for fermentation, and it was found that 36 h is an optimum time for the effective production of oligosaccharides. Subsequently, Bio-Gel P-10 based size exclusion resin was suitable for the oligosaccharides’ separation. Oligosaccharide fractions were further screened for their monosaccharide composition and structural analysis. It was confirmed that these oligosaccharides contained various acidic and neutral monosaccharides with varying proportions in different fractions. The FT-IR spectroscopy confirmed the presence of characteristic glycosidic linkages. Our results showed that the oligosaccharide fractions had strong in vitro antioxidant activity by scavenging the DPPH and H_2_O_2_ free radicals and that the activity was comparable to the ascorbic acid and gallic acid used as standard antioxidants. Further, it was noted that oligosaccharides exhibited remarkable hepatoprotective activity against Cr(VI)-induced hepatotoxicity in the mice model. Cr exposure in the mice led to a severe disturbance in the antioxidant defense system, biochemical indices, and histopathological parameters. However, oligosaccharides supplementation attenuated the oxidative disturbance and restored liver damage in most of the measured parameters in mice. Therefore, it can be concluded that oligosaccharides have a powerful role in mitigating the Cr(VI) induced toxicity by reducing the production of free radicals, potentiating the antioxidant defense system, and reverting the apoptotic damages.

## Figures and Tables

**Table 1:** Mono- and disaccharide composition of oligosaccharides fractions obtained through microbial digestion of cress seed mucilage polysaccharides.

	Saccharides	DF53	DF62	DF72	DF73
Neutral Saccharides	Arabinose	6.53	13.17	2.78	2.98
	Fucose	3.67	5.12	2.09	0.85
	Xylose	Traces	4.32	1.18	Traces
	Galactose	4.98	5.38	3.19	3.17
	Glucose	17.32	31.45	17.56	5.23
	Rhamnose	10.64	-	3.03	4.98
	Sucrose	5.67	9.28	5.23	3.16
	Maltose	3.28	0.7	6.98	2.67
Acidic Monosaccharaides	Galacturonic Acid	32.18	26.34	50.83	71.23
	Glucuronic Acid	15.63	4.24	7.13	5.43

**Table 2:** Half-maximal inhibitory concentrations (IC_50_) in µg/mL of the oligosaccharide fractions against free radicals, where ascorbic acid served as standard drug against DPPH and gallic acid against H_2_O_2_ free radical.

Half-Maximal Inhibitory Concentration (IC_50_) in µg/mL		
	DPPH	H_2_O_2_
EPP	93.1 ± 1.65	80.77 ± 0.01
DF53	55.7 ± 2.78	55.56 ± 0.36
DF62	61.91 ± 1.98	59.46 ± 5.32
DF72	40.92 ± 3.25	59.07 ± 4.01
DF73	23.28 ± 2.88	51.63 ± 4.77
Standard Drug	22.01 ± 4.76	29.14 ± 2.28

## Data Availability

The original contributions presented in the study are included in the article, and further inquiries can be directed to the corresponding authors.

## Abbreviations

ALP	Alkaline Phosphatases
ALT	Alanine Aminotransferase
AST	Aspartate Aminotransferase
CFU/mL	Colony Forming Unit per Milliliter
Cr	Chromium
DPPH	1,1-diphenyl-2-picrylhydrazyl
EPP	Ethanol Precipitated Polysaccharides
FT-IR	Fourier Transformed Infrared
GC-MS	Gas Chromatography-Mass Spectrometry
GPC	Gel Permeation Chromatography
H_2_O_2_	Hydrogen Peroxide
HPLC	High-Performance Liquid Chromatography
IC_50_	Half-Maximal Inhibitory Concentration
LPO	Lipid Peroxidation
MDA	Malondialdehyde
ROS	Reactive Oxygen Species
TCC	Total Carbohydrates Content
YPD	Yeast Extract, Peptone, and Dextrose
