# Correction: Khan et al. Anti-Oxidative and Anti-Apoptotic Oligosaccharides from *Pichia pastoris*-Fermented Cress Polysaccharides Ameliorate Chromium-Induced Liver Toxicity. *Pharmaceuticals* 2024, *17*, 958

**DOI:** 10.3390/ph18030365

**Published:** 2025-03-04

**Authors:** Imdad Ullah Khan, Aqsa Aqsa, Yusra Jamil, Naveed Khan, Amjad Iqbal, Sajid Ali, Muhammad Hamayun, Abdulwahed Fahad Alrefaei, Turki Kh. Faraj, Bokyung Lee, Ayaz Ahmad

**Affiliations:** 1 Department of Biotechnology, Abdul Wali Khan University Mardan, Mardan 23200, Pakistan; ik16092@gmail.com (I.U.K.); aqsaa3056@gmail.com (A.A.); yusrajamil22@gmail.com (Y.J.); naveedkhan@awkum.edu.pk (N.K.); 2 Department of Food Science and Technology, Abdul Wali Khan University Mardan, Mardan 23200, Pakistan; amjadiqbal@awkum.edu.pk; 3 Department of Horticulture and Life Science, Yeungnam University, Gyeongsan 38541, Republic of Korea; 4 Department of Botany, Abdul Wali Khan University Mardan, Mardan 23200, Pakistan; hamayun@awkum.edu.pk; 5 Department of Zoology, College of Science, King Saud University, Riyadh 145111, Saudi Arabia; afrefaei@ksu.edu.sa; 6 Department of Soil Science, College of Food and Agriculture Sciences, King Saud University, Riyadh 145111, Saudi Arabia; talasiri@ksu.edu.sa; 7 Department of Health Sciences, The Graduate School of Dong-A University, Busan 49315, Republic of Korea; bolee@dau.ac.kr

## Error in Figure

In the original publication [1], there was a mistake in Figure 10 as published. Figure 10 was intended to display images representing different experimental conditions. However, upon review, I noticed that image E and image H are identical, which appears to have occurred due to a mistake during figure preparation. Image E was inadvertently duplicated instead of including the correct image representing its experimental condition. This correction ensures the accurate representation of all experimental conditions. 

The corrected Figure 10 appears below. The authors state that the scientific conclusions are unaffected. This correction was approved by the Academic Editor. The original publication has also been updated.

**Figure 10:**
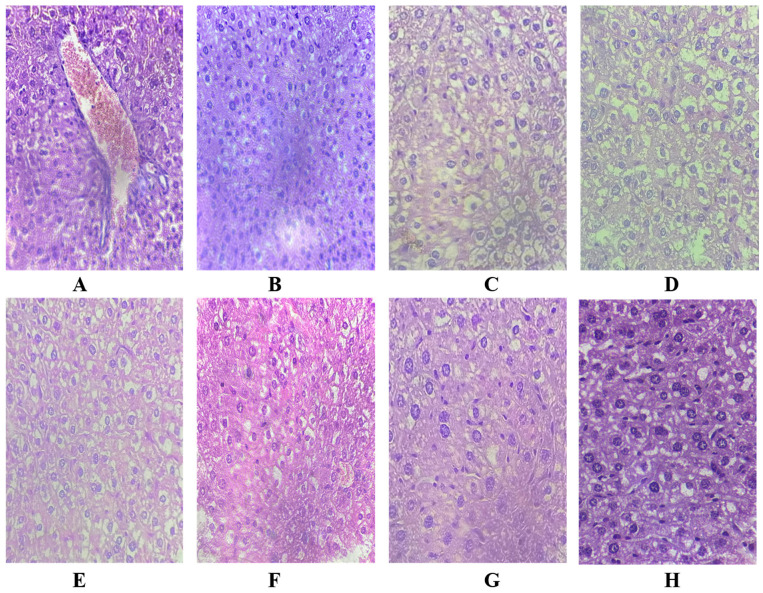
Histology of the mice liver stained with H&E stain: (**A**) received 100 mg/kg of Cr(VI) only, (**B**,**C**) normal control, (**D**) received 100 mg/kg of Cr(VI) + 200 mg/kg of DF53, (**E**) received 100 mg/kg of Cr(VI) + 200 mg/kg of DF62, (**F**) received 100 mg/kg of Cr(VI) + 200 mg/kg of DF72, (**G**) received 100 mg/kg of Cr(VI) + 200 mg/kg of DF73 and (**H**) received 100 mg/kg of Cr(VI) + 100 mg/kg of ascorbic acid.
